# Plasma inflammatory biomarkers in cerebral small vessel disease: A review

**DOI:** 10.1111/cns.14047

**Published:** 2022-12-07

**Authors:** Shuling Wan, Chaitu Dandu, Guangyu Han, Yibing Guo, Yuchuan Ding, Haiqing Song, Ran Meng

**Affiliations:** ^1^ Department of Neurology, National Center for Neurological Disorders, Xuanwu Hospital Capital Medical University Beijing China; ^2^ Advanced Center of Stroke Beijing Institute for Brain Disorders Beijing China; ^3^ Department of Neurosurgery Wayne State University School of Medicine Detroit Michigan USA

**Keywords:** cerebral small vessel disease, endothelial dysfunction, imaging features, inflammatory biomarkers

## Abstract

Cerebral small vessel disease (CSVD) is a group of pathological processes affecting small arteries, arterioles, capillaries, and small veins of the brain. It is one of the most common subtypes of cerebrovascular diseases, especially highly prevalent in elderly populations, and is associated with stroke occurrence and recurrence, cognitive impairment, gait disorders, psychological disturbance, and dysuria. Its diagnosis mainly depends on MRI, characterized by recent small subcortical infarcts, lacunes, white matter hyperintensities (WMHs), enlarged perivascular spaces (EPVS), cerebral microbleeds (CMBs), and brain atrophy. While the pathophysiological processes of CSVD are not fully understood at present, inflammation is noticed as playing an important role. Herein, we aimed to review the relationship between plasma inflammatory biomarkers and the MRI features of CSVD, to provide background for further research.

## Graphical abstract

Existing evidence indicates that inflammation may play an important part in the pathogenesis of CSVD. The review focused on the association between inflammatory biomarkers and MRI features of CSVD, to make a reference for further research.

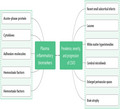

## 1 INTRODUCTION

Cerebral small vessel disease (CSVD) is a group of pathological processes affecting small arteries, arterioles, capillaries, and small veins of the brain, with a variety of etiologies.^1^ It is very common and highly prevalent in the elderly, responsible for lacunar stroke (LS), vascular dementia (VaD), and vascular parkinsonism (VaP).^2^ The symptoms of CSVD in some patients are not remarkable, or will present as stroke,^3^, ^4^, ^5^ cognitive impairment,^6^, ^7^, ^8^, ^9^, ^10^, ^11^, ^12^, ^13^, ^14^, ^15^, ^16^ gait disorders,^11^, ^17^ psychological disturbance (such as depression, delirium, and apathy),^17^, ^18^ urinary disorders, and ultimately loss of independence and disability, all of which place a heavy burden on both family and society.^19^, ^20^ The features of CSVD on magnetic resonance imaging (MRI) mainly include recent small subcortical infarcts, lacunes, white matter hyperintensities (WMHs), enlarged perivascular spaces (EPVS), cerebral microbleeds (CMBs), and brain atrophy.^21^

Age and genetic factors are nonmodifiable risk factors for CSVD. The prevalence of CSVD increases with increasing age.^22^, ^23^, ^24^ Currently, several monogenic inherited CSVDs have been identified, including cerebral autosomal dominant arteriopathy with subcortical infarcts and leukoencephalopathy (CADASIL), cerebral autosomal recessive arteriopathy with subcortical infarcts and leukoencephalopathy (CARASIL), cathepsin A‐related arteriopathy with strokes and leukoencephalopathy (CARASAL), hereditary diffuse leukoencephalopathy with spheroids (HDLS), COL4A1/2‐related disorders, and Fabry disease.^25^, ^26^ In addition, the most important modifiable risk factor is arterial hypertension, and other risk factors include diabetes mellitus, hyperlipidemia, smoking,^27^ obstructive sleep apnea,^28^ as well as socioeconomic and education status.^29^

Currently, the pathogenesis of CSVD is not completely understood; however, endothelial dysfunction and subsequently increased blood–brain barrier (BBB) permeability have been implicated in the development of CSVD, supported by experimental studies,^30^, ^31^, ^32^ neuropathological studies,^33^, ^34^, ^35^ and imaging studies.^36^, ^37^, ^38^, ^39^ In addition, systemic inflammation was found to induce the migration of brain‐resident microglia to cerebral vasculature. These microglia were found to initially maintain BBB integrity, but then phagocytose astrocytic end‐feet and lead to BBB dysfunction.^40^ Previous studies have shown that markers of systemic inflammation and vascular inflammatory/endothelial dysfunction may be associated with the prevalence, severity, and progression of CSVD. Herein, we aimed to review the research progress of the association between inflammatory biomarkers and MRI features of CSVD, to make a reference for further research.

## 2 METHODS

We searched PubMed and EMBASE databases by using the following search items: (cerebral small vessel disease) OR (CSVD) OR (leukoaraiosis) OR (white matter lesions) OR (white matter hyperintensities) OR (lacunar stroke) OR (lacunes) OR (perivascular spaces) OR (microbleeds) AND (biomarkers) OR (inflammation) OR (blood–brain barrier) OR (endothelial dysfunction). No language restrictions were applied. In addition, the references of the retrieved articles were reviewed and included if appropriate. The final selection was based on relevance, as judged by the authors.

After a careful reading of the retrieved literatures, we obtained a series of plasma biomarkers related to MRI features of CSVD, including systemic inflammation markers, such as C‐reactive protein (CRP), serum amyloid‐A protein (SAA), fibrinogen and cytokines (interleukin [IL], tumor necrosis factor [TNF], osteoprotegerin [OPG] and vascular endothelial growth factor [VEGF]), and vascular inflammation markers, such as adhesion molecules (E‐selectin, P‐selectin, intercellular adhesion molecule‐1 [ICAM‐1], and vascular cell adhesion molecule‐1 [VCAM‐1]), hemostasis factors (thrombomodulin [TM], tissue factor [TF], tissue factor pathway inhibitor [TFPI], von Willebrand factor [vWF], prothrombin fragment 1 + 2 [F 1 + 2], thrombin‐antithrombin complex [TAT], D‐dimer, tissue‐type plasminogen activator [t‐PA] and plasminogen activator inhibitor‐1 [PAI‐1]), and homocysteine (Hcy).

## 3 RESULTS

### 3.1 Imaging features of CSVD

**FIGURE 1:**
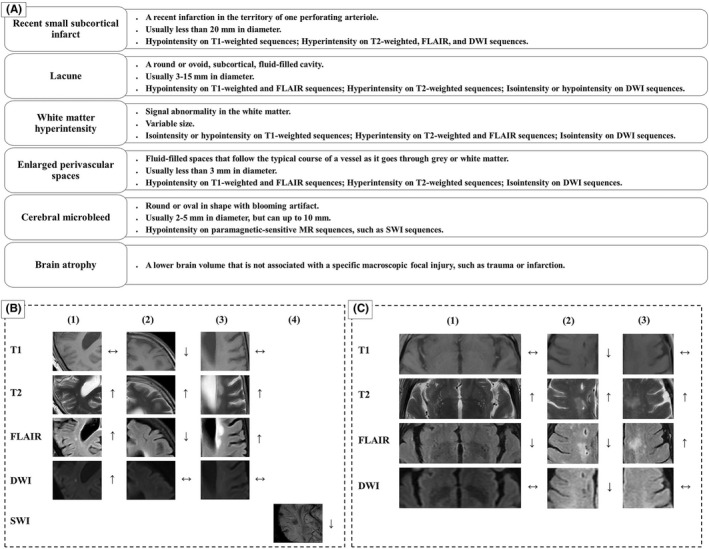
Features of CSVD on MRI. (A) The features of CSVD on MRI mainly included recent small subcortical infarcts, lacunes, white matter hyperintensities, enlarged perivascular spaces, cerebral microbleed, and brain atrophy. (B) Cranial MRI findings in a 56‐year‐old female with vascular dementia: (1) recent small subcortical infarct, (2) lacune, (3) white matter hyperintensities, and (4) cerebral microbleeds. (C) Cranial MRI findings in a 66‐year‐old male with clinical presentation of bradykinesia: (1) enlarged perivascular spaces, (2) lacunes, and (3) white matter hyperintensities. DWI represents diffusion‐weighted imaging; FLAIR, fluid‐attenuated inversion recovery; SWI represents susceptibility‐weighted imaging.

Currently, the clinical diagnosis of CSVD mainly relies on imaging techniques, such as CT and MRI. The features of CSVD on MRI mainly include recent small subcortical infarcts, lacunes, WMHs, EPVS, CMBs, and brain atrophy (Figure 1).^21^ A systematic review and meta‐analysis showed that there were statistically significant associations between WMHs and incident ischemic or hemorrhagic stroke, all‐cause dementia, depression as well as all‐cause mortality, with hazard ratios ranged 1.27–2.32. Significant associations were also found between other MRI features (lacunes, CMBs, and brain atrophy) and several aforementioned clinical events.^41^ Based on the MRI features, Park and colleagues classified CSVD into three distinct classes: multiple, small, deep WMHs with a low burden of lacunes and CMBs (class I); large periventricular WMHs with a high burden of lacunes and CMBs (class II); and limited juxtaventricular WMHs lacking lacunes and CMBs (class III). The authors found that class II was associated with older age, diabetes, and higher neutrophil‐to‐lymphocyte ratio (NLR); while, class I was related to smoking and higher uric acid levels.^42^ However, because of the limitation of cross‐sectional design, the outcomes of each class are unclear.

More advanced imaging methods, including diffusion tensor imaging (DTI), vessel wall imaging (VWI) and ultra‐high‐field MRI (such as 7‐T MRI), can detect microstructure damage earlier than conventional MRI sequences, providing the possibility for a reversal in early stage. DTI is a proper quantitative method to assess microstructure integrity of white matter (WM), including WMH and normal‐appearing WM [NAWM]. A nine‐year longitudinal study showed that DTI could detect impaired WM microstructure integrity years before WMH become visible on conventional neuroimaging. DTI‐derived parameters, such as fractional anisotropy (FA) and mean diffusivity (MD), have been shown to be associated with cognitive impairment, gait disorders, risk of mortality, and CSVD severity.^43^ However, Brandhofe and colleagues found that the T2 relaxation time of NAWM was associated with cognition in CSVD patients, which the FA and MD of the NAWM failed to achieve.^44^ More recently, newer acquisitions and models such as the free‐water (FW) model have shown greater associations with WM injury and cognitive impairment, as compared to the traditional DTI measures.^43^ The development of vessel wall imaging (VWI) allows detailed evaluation of the vessel wall and lumen in a single image setting, enabling the visualization of actual pathology of the vessel wall before luminal narrowing becomes apparent, may help to depict the mechanisms underlying CSVD. Furthermore, this MRI technique may benefit from 7‐T MRI. A study using 7‐T vessel wall MRI showed that a higher intracranial atherosclerosis (ICAS) burden was associated with more CSVD MRI features.^45^ In addition, 7‐T MRI has been shown to be more sensitive in detecting cerebral microinfarcts (CMIs), a manifestation of both small vessel and large vessel disease that is independently associated with cognitive impairment, as compared to conventional MRI.^46^

### 3.2 Biomarkers of inflammation in CSVD

#### 3.2.1 Systemic inflammation markers

##### C‐reactive protein

C‐reactive protein (CRP) belongs to the pentraxin family of calcium‐dependent ligand‐binding plasma proteins, which is the first described acute‐phase protein and an exquisitely sensitive but nonspecific systemic marker of inflammation and tissue damage.^47^ CRP synthesis is predominantly in hepatocytes and principally regulated by the pro‐inflammatory cytokines, most notably IL‐6 and to a lesser degree IL‐1 and tumor necrosis factor alpha (TNF‐α).^48^ Current evidence suggests that plasma CRP level has a direct effect on endothelial cells (ECs) by decreasing endothelial nitric oxide synthase (eNOS) expression and bioactivity, reducing nitric oxide (NO) production and increasing the release of vasoconstrictors and adhesion molecules, such as ICAM‐1, VCAM‐1, and E‐selectin, thus playing an important part in endothelial dysfunction.^49^, ^50^ Newer high‐sensitivity assays can measure CRP levels in the 0.01–10 mg/L range, that is hs‐CRP, and can therefore quantify low‐grade systemic inflammation in the absence of obvious systemic inflammation or immune disorders.^51^, ^52^

##### Serum amyloid‐A protein

Serum amyloid‐A protein (SAA) is also an acute‐phase protein synthesized by hepatocytes in response to cytokine activation, with sensitivity, response speed, and dynamic range comparable to CRP.^47^ SAA is a marker of systemic inflammation by stimulating vascular cells to express cytokines, chemokines, adhesion molecules (such as ICAM‐1, VCAM‐1, and E‐selectin), and matrix metalloproteinases (MMPs).^53^

##### Fibrinogen

Fibrinogen is a glycoprotein consisting of three pairs of disulfide‐bonded polypeptide chains, namely Aα, Bβ, and γ. It is synthesized primarily in hepatocytes and has a plasma half‐life of 3–4 days in humans.^54^ Fibrinogen is also an acute‐phase protein with no specificity. Its level appears to be genetically determined, with circadian and seasonal variations, and is influenced by a number of circumstances and medications. Inflammation can elicit the release of fibrinogen in peripheral blood.^55^ The normal blood level of fibrinogen is about 2 mg/ml, but it can rise to 3.6–4 mg/ml during different cardiovascular diseases.^56^ Elevated levels of fibrinogen can lead to increased plasma viscosity and erythrocyte aggregation, enhanced platelet thrombogenesis and vascular reactivity, and disruption of EC layer integrity, resulting in vascular dysfunction.^54^, ^56^, ^57^, ^58^, ^59^

##### Cytokines

The cytokine family consists of a variety of proteins that play an important role in inflammation. IL‐6 is an upstream inflammatory cytokine that leads to the production of downstream acute‐phase reactant in the liver and plays a central role in the inflammatory response.^60^ TNF‐α is a pleiotropic cytokine synthesized by a number of cell types and plays an important role in inflammatory response through two cell membrane receptors, TNFR1 and TNFR2.^61^, ^62^ This pro‐inflammatory and tissue‐destructive cytokine is toxic to oligodendrocytes, thus mediating myelin damage and white matter degeneration.^61^ Osteoprotegerin (OPG) is a soluble glycoprotein belonging to the tumor necrosis factor receptor superfamily.^62^, ^63^ It is a key regulator of immune defense system, apoptosis, and vascular calcification in human vascular biology. It binds to the receptor activator of NF kappa B ligand (RANKL) and tumor necrosis factor‐related apoptosis‐inducing ligand (TRAIL), thereby inhibiting activation of their pro‐inflammatory and proapoptotic signal pathway.^63^ Vascular endothelial growth factor (VEGF) is the most specific growth factor of ECs and known as the main inducer of angiogenesis.^64^, ^65^ Animal studies showed that increased VEGF expression was associated with increased BBB permeability, resulting in vasogenic edema and leakage of blood‐derived substances into the brain parenchyma.^65^, ^66^

#### 3.2.2 Vascular inflammation and endothelial dysfunction markers

##### Adhesion molecules

Vascular endothelial activation plays a key role in inflammation‐related pathological states. Aberrant or persistent activation of local vascular endothelium triggered by cytokine signaling can lead to overexpression of adhesion molecules, which in turn facilitate the recruitment, adhesion, and infiltration of leukocytes, thereby damaging vasculature and localized tissues.^67^ Endothelial activation and dysfunction can be evaluated in vivo by measuring the levels of soluble plasma markers released into the blood when ECs are activated by a variety of stimulus.^68^ E‐ and P‐selectins are members of the selectin family of adhesion molecules and facilitate leukocytes rolling along and reversible adhesion to vascular endothelium, whereas ICAM‐1 and VCAM‐1 belong to the immunoglobulin gene superfamily and mediate the adhesion, activation, and transendothelial migration of leukocytes.^62^, ^67^, ^68^, ^69^

##### Hemostasis factors

Similarly, thrombomodulin (TM) is normally expressed on the surface of ECs where it regulates protein C activity in conjunction with thrombin. Elevated plasma TM levels are thought to reflect endothelial injury.^70^ Tissue factor (TF) can be expressed on monocytes and ECs in response to the stimulation of factors such as TNF. TF triggers the extrinsic coagulation pathway by binding to factor VII, thus increased levels of soluble TF reflect prothrombotic change.^70^ Tissue factor pathway inhibitor (TFPI) is the physiological inhibitor of TF, which binds to factor Xa within the TF‐VIIa‐Xa complex, thereby preventing thrombosis.^70^ The von Willebrand factor (vWF) is only synthesized in ECs and megakaryocytes, most of which are stored in platelet α‐granules and endothelial Weibel–Palade bodies (WPBs). Plasma vWF is majorly derived from ECs.^71^ The vWF can be released into plasma and basement membrane on endothelial activation, thus to a certain extent reflecting endothelial activation.^72^ On the contrary, vWF is a large multimeric glycoprotein in plasma, which plays a key role in hemostasis and thrombosis by mediating the adhesion of platelet to damaged and activated blood vessels.^71^ Tissue‐type plasminogen activator (t‐PA) and plasminogen activator inhibitor‐1 (PAI‐1) determine the balance of fibrinolytic activity, both of which are synthesized and secreted from ECs.^73^

##### Homocysteine

Another widely investigated inflammatory marker is homocysteine (Hcy), a thiol‐containing amino acid generated during the metabolization of methionine which is a natural amino acid consumed through normal diet.^74^ There are three pathways involved in the Hcy metabolism, the remethylation pathway, the transsulfuration pathway, and direct release into the extracellular fluid.^75^ Genetic variations of critical enzymes (e.g., Methyleneterahydrofolate reductase [MTHFR], methionine synthase [MS], methionine synthase reductase [MTRR], and cystathionine‐beta‐synthase [CBS]) in Hcy metabolism, deficiency of B‐vitamins (B2, B6, B9, and B12), unhealthy living habit (smoking and alcoholism), renal failure, and medications can lead to Hcy accumulation and increase of plasma Hcy levels.^76^ There is evidence that elevated Hcy levels can damage ECs through a variety of pathological pathways, including vasomotor dysfunction, oxidative stress, vascular inflammation, prothrombotic condition, cellular hypomethylation, and protein N‐homocysteinylation, among which vascular inflammation plays an important role.^75^, ^77^, ^78^, ^79^, ^80^, ^81^, ^82^, ^83^

### 3.3 Association between inflammatory biomarkers and CSVD

#### 3.3.1 CRP in CSVD

##### CRP and CSVD prevalence

There is a number of evidence showed that CRP was associated with CSVD (Table 1). Hoshi et al. showed an association between the hs‐CRP levels and the presence of silent brain infarction (SBI) in one hundred ninety‐four patients without histories of cardiovascular accidents. In unadjusted analysis, each standard deviation (SD) increase in log hs‐CRP was associated with 1.85‐fold higher likelihood for SBI. After adjustments for age, sex, traditional cardiovascular risk factors, medication use, and mean carotid intima–media thickness (IMT), the association persisted.^84^ Similarly, in older Japanese with hypertension, the subjects with SBI had significantly higher hs‐CRP levels than those without after adjusting for confounding factors, and the odds ratio (OR) for the presence of SBI was increased with the quartile of hs‐CRP levels.^85^ Sefuri study indicated that hs‐CRP was associated with confluent but not punctate deep white matter hyperintensity (DWMHs) in 259 community‐dwelling older adults with a mean age of 68.4 years. Logistic regression analysis showed that the log_10_ hs‐CRP value was one of the independent predictors of confluent DWMHs (OR = 3.024; 95% confidence interval [CI], 1.305–7.008; *p* = 0.010). Path analysis based on structural equation modeling (SEM) revealed that the direct path from log_10_ hs‐CRP to DWMHs was significant (*β* = 0.119, *p* = 0.039).^18^ In 206 patients with first acute LS, Koh et al. showed that CRP levels were significantly higher in patients with microbleeds than those without (0.93 ± 0.97 vs. 0.52 ± 0.31, *p* = 0.047).^86^ The role of CRP in the development of CSVD may vary among ethnic groups. Fornage et al. investigated the association between plasma CRP level, common variation in the CRP gene, and presence of WMH and brain infarcts (BI) in the elderly of the Cardiovascular Health Study and found a significant association between higher level of plasma CRP and higher risk of WMH in Whites, but not in Blacks. Adjustment for confounding factors did not significantly attenuate this association.^87^

**TABLE 1:** Associations of systemic inflammation biomarkers with MRI features of CSVD

Source	Study design	No.	Filed strength	MRI features	CRP	Fib	IL‐6	TNF‐α	TNFR2	OPG	VEGF
Hoshi et al.^84^	Cross‐sectional	194	1.5 T	Lacunes	OR = 1.5		OR = 1.85				
Ishikawa et al.^85^	Cross‐sectional	514	1.5 T	Lacunes	OR = 2.08						
				DWMH	No						
Yao et al.^18^	Cross‐sectional	259	1.5 T	cDWMH	OR = 2.88						
Koh et al.^86^	Cross‐sectional	206	1.5 T	CMBs	Yes						
Fornage et al.^87^	Cross‐sectional	3644	1.5 T	WMHs	Yes		Yes				
				BI	Yes		Yes				
Wada et al.^88^	Cross‐sectional	689	‐	Lacunes	No						
				WMHs	No						
Wright et al.^89^	Cross‐sectional	527	1.5 T	WMHs	No						
Mitaki et al.^90^	Cross‐sectional	519	1.5 T	Lacunes	OR = 3.57						
				WMHs	No						
				CMBs	No						
Hilal et al.^92^	Cross‐sectional	2814	1.5 T	Lacunes	RR = 1.61						
				WMHs	*β* = 0.06						
				CMBs	RR = 0.74						
				EPVS	RR = 1.01						
Schmidt et al.^93^	Longitudinal (6‐year)	505	1.5 T	Lacunes	No						
				WMHs	No						
van Dijk et al.^94^	Longitudinal (3.3‐year)	636	1.5 T	Lacunes	No						
				PVH	OR = 3.1						
				DWMH	OR = 2.5						
Umemura et al.^95^	Longitudinal (6‐year)	190	1.5 T	Lacunes	OR = 1.54 (3‐year)						
				WMHs	No						
Wada et al.^97^	Cross‐sectional	667	‐	Lacunes	No	No					
				WMHs	No	OR = 1.99					
Wei et al.^98^	Cross‐sectional	186	3.0 T	PVH		OR = 12.6					
				DWMH		No					
You et al.^99^	Cross‐sectional	170	3.0 T	PVH		OR = 2.1					
				DWMH		OR = 1.8					
Guo et al.^100^	Cross‐sectional	74	3.0 T	WMHs	No	OR = 1.1					
Shen et al.^101^	Cross‐sectional	164	3.0 T	EPVS		No					
Liu et al.^102^	Cross‐sectional	85	3 T	CMBs		OR = 2.16					
Staszewski et al.^103^	Longitudinal (2‐year)	123	1.5 T	Lacunes		OR = 1.02					
				WMHs		OR = 1.01					
Jefferson et al.^104^	Cross‐sectional	1926	1.0 T	WMHs	No		No	No	No	No	
Satizabal et al.^105^	Longitudinal (4‐year)	1841	1.5 T	Lacunes	No		No				
				WMHs	No		No				
Satizabal et al.^106^	Cross‐sectional	1820	1.5 T	BG‐EPVS	No		OR = 1.38				
				WM‐EPVS	No		No				
Staszewski et al.^2^	Longitudinal (2‐year)	123	1.5 T	Lacunes	No		No	No			
				WMHs	No		No	No			
Noz et al.^107^	Longitudinal (2‐year)	51	1.5 T	WMHs	No		*r* = 0.29				
Shoamanesh et al.^62^	Cross‐sectional	1763	1.0 T	CMBs	No	No	No	No	OR = 2.2	No	No
				SCI/WMHs	No	No	No	No	No	OR = 1.1	No
Zhang et al.^65^	Cross‐sectional	146	3.0 T	CMBs							OR = 2.37
Rouhl et al.^19^	Cross‐sectional	346	1.5 T	Lacunes	No						
				WMHs	No						
				CMBs	No						
				BG‐EPVS	No						
Kawamura et al.^114^	Longitudinal (3‐year)	120	1.5 T	Lacunes	No						
Kario et al.^115^	Cross‐sectional	160	1.5 T	Lacunes		Yes					
Gottesman et al.^116^	Cross‐sectional	410	1.5 T	Lacunes	No	No					
Wang et al.^117^	Cross‐sectional	100	1.5 T	BG‐EPVS	No	No	No	No			

Abbreviations: BG‐EPVS, basal ganglia‐EPVS; BI, brain infarct; cDWMH, confluent DWMH; CMB, cerebral microbleed; CRP, C‐reactive protein; DWMH, deep WMH; EPVS, enlarged perivascular spaces; Fib, fibrinogen; IL, interleukin; MCP‐1, monocyte chemoattractant protein‐1; OPG, osteoprotegerin; OR, odds ratio; PVH, periventricular hyperintensity; RR, rate ratio; SCI, silent cerebral infarct; TNFR2, tumor necrosis factor receptor‐2; TNF‐α, tumor necrosis factor‐α; VFGF, vascular endothelial growth factor; WM‐EPVS, white matter‐EPVS; WMH, white matter hyperintensity; *β*/*r*, correlation coefficient.

##### CRP and CSVD severity

In a community‐based group of Japanese elderly (*n* = 689), there was no statistical significance between hs‐CRP and the number of lacunes or grades of WMHs.^88^ Furthermore, Wright et al. showed that participants with hs‐CRP in the upper quartile had larger volume of WMH after adjustment for sociodemographic and vascular risk factors, whereas, further adjusting for all biomarkers simultaneously, hs‐CRP was not associated with the volume of WMH.^89^ However, considerable evidence showed that the CRP levels were associated with CSVD severity: Mitaki et al. found that subjects with higher hs‐CRP had more lacunar infarcts (*p* = 0.02) and CMBs (*p* = 0.03), and more severe DWMH (*p* = 0.04) and periventricular hyperintensity (PVH) (*p* = 0.04). Logistic regression analysis showed that the association between the tertiles of hs‐CRP and the presence of lacunar infarcts was significant after adjustment for traditional cardiovascular risk factors.^90^ Walker et al. further investigated the association between 21‐year longitudinal pattern of hs‐CRP and the development of the volume of WMH. After controlling for demographic variables and cardiovascular risk factors, participants in the early ascending group (low CRP [<3 mg/L] at visit 2, and elevated CRP [3 mg/L] at visits 4 and 5) demonstrated greater volume of WMH (0.35 SD; 95% CI: 0.07–0.64; *p* = 0.015) compared to those in stable low group (low CRP at all 3 visits).^91^ Hilal et al. showed that higher CRP levels were associated with larger WMHV and increasing numbers of lacunes, EPVS, and deep/infratentorial microbleeds in 2814 participants with a mean age of 56.9 years from the Rotterdam Study; additionally, higher CRP levels were found to be associated with smaller gray matter volume, but not with white matter and hippocampal volumes.^92^

##### CRP and CSVD progression

Results from the studies of CRP in CSVD progression are conflicting. Using prospective data from a large random sample of middle‐aged and elderly, asymptomatic, community‐dwelling subjects in the Austrian Stroke Prevention Study, Schmidt et al. demonstrated no significant association between the levels of CRP and the severity or progression of WMHs or lacunes.^93^ A study based on the prospective, population‐based Rotterdam Scan confirmed that higher levels of CRP were significantly associated with the presence and progression of WMHs, even after adjusted cardiovascular risk factors and carotid atherosclerosis. In addition, participants with higher CRP levels tended to have more prevalent and incident lacunar infarcts than those with lower CRP levels; however, these associations were not significant.^94^ In a 6‐year longitudinal study with 190 type 2 diabetic patients with a mean age of 62.7 years, higher baseline levels of hs‐CRP were significantly associated with SBI progression at the year‐3 follow‐up (*p* = 0.020), but not the year‐6 follow‐up. Whereas no significant association of baseline hs‐CRP levels with year 3 or 6 of WMH progression was observed.^95^

#### 3.3.2 SAA and CSVD

The relationship between SAA and MRI features of CSVD is unclear yet. The ongoing LIMITS (Levels of Inflammatory Markers in the Treatment of Stroke) study may provide the identification of inflammatory biomarkers, including hs‐CRP, SAA, IL‐6, CD40 ligand (CD40L), TNFR1, and monocyte chemoattractant protein‐1 (MCP‐1), for using in predicting recurrent stroke and other vascular events among patients with a history of small vessel IS or lacunes.^96^

#### 3.3.3 Fibrinogen and CSVD

The relationship between fibrinogen and CSVD has already been investigated (Table 1). A cross‐sectional study showed that in community‐based Japanese elderly, plasma fibrinogen levels were associated with more lacunes and higher grades of WMHs, and there was an independent association between fibrinogen and WMHs. Additionally, subjects with high levels of fibrinogen accompanied by high levels of vWF or TM were more likely to present with moderate WMHs.^97^ Wei et al. showed that in patients with IS and atrial fibrillation (AF), plasma fibrinogen levels were independently and positively correlated with the presence of WMHs and PVH (both *p* < 0.05). Compared with patients with normal level of fibrinogen, those with abnormally high levels of fibrinogen had an increased tendency for the presence of WMHs and PVH, with OR of 14.037 (95% CI 2.588–76.131) and 12.567 (95% CI 2.572–61.395), respectively. However, there was no significant difference in fibrinogen levels between patients with and without DWMH.^98^ By contrast, You et al. demonstrated that in nondiabetic patients with noncardiogenic acute IS, fibrinogen was independently and positively associated with WMHs, regardless of PVH and DWMH, with OR of 2.114 (95% CI 1.034–4.322) and 1.788 (95% CI 1.170–2.732), respectively.^99^ Furthermore, a cross‐sectional study indicated that fibrinogen was an independent risk factor for the severity of WMHs in CADASIL patients, with the OR of 1.064 (95% CI 1.004–1.127), but not in sporadic CSVD (sCSVD) patients.^100^ Likely, study by Shen et al. showed that the fibrinogen level was associated with the degrees of white matter (WM)‐EPVS (*p* = 0.018), but not with the degrees of basal ganglia (BG)‐EPVS (*p* = 0.347). Additionally, the association between fibrinogen level and WM‐EPVS disappeared after adjustment.^101^ Liu and colleagues revealed that the presence of CMBs in IS patients with AF and/or rheumatic heart disease was independently associated with elevated fibrinogen levels (OR 2.16, 95% CI 1.20–3.90, *p* = 0.01).^102^ Notably, a 2‐year, single‐center, prospective, cohort study indicated that fibrinogen was significantly correlated with an increased risk of new lacunes or WMHs progression, with OR of 1.02 (95% CI 1.006–1.011), regardless of the clinical SVD manifestation (LS, VaD and VaP).^103^

#### 3.3.4 IL‐6 and CSVD

##### IL‐6 and CSVD prevalence

The relationship between IL‐6 and CSVD remains controversial (Table 1). Jefferson et al. found that IL‐6 levels were not significantly related to white matter hyperintensities /total cranial volume (WMH/TCV) in 1926 Framingham Offspring participants free from clinical stroke, transient ischemic attack (TIA), or dementia with a mean age of 60 years.^104^ Fornage et al. observed significant and graded associations between plasma IL6 levels and WMH in both Whites and Blacks and between plasma IL6 levels and BI in Whites. Additionally, the study provided evidence of a genetic basis underlying the association between plasma biomarkers of inflammation and CSVD. Common haplotypes of the IL6 gene were found to be significantly associated with WMH and BI in Whites, but not in Blacks.^87^ Hoshi et al. showed that in 194 patients without histories of cardiovascular accidents, higher IL‐6 levels were associated with higher likelihood for SBI (OR, 2.00/SD increase), and this association was slightly attenuated after adjustment for traditional cardiovascular risk factors and carotid IMT (OR, 1.85/SD increase).^84^

##### IL‐6 and CSVD severity

Satizabal et al. studied 1841 participants aged 65–80 years from the Three City‐Dijon cohort and showed that higher levels of IL‐6 were cross‐sectionally associated with higher total and periventricular WMH volume (WMHV), independent of age, sex, and vascular risk factors. However, there was no association between IL‐6 levels and deep WMH. Additionally, participants with elevated IL‐6 levels were more likely to have SBI, although nonsignificantly.^105^ Similarly, the authors also demonstrated that elevated IL‐6 levels were significantly associated with the burden of BG‐EPVS.^106^

##### IL‐6 and CSVD progression

Satizabal and colleagues observed no associations between baseline plasma IL‐6 levels and the evolution of CSVD MRI findings over 4 years in the Three City‐Dijon cohort study.^105^ By contrast, Staszewski et al. showed that after adjustment for age, sex, baseline mean arterial pressure (MAP), and MRI lesions load, IL‐6 was significantly associated with the risk of any radiological progression (OR, 7.4; 95% CI, 1.48–37; *p* = 0.02), which was defined as an increase in WMHs or the development of new lacunes in one or more periventricular and/or subcortical regions. Besides, IL‐6 was marginally associated with the development of new lacunes (OR, 6.0; 95% CI, 0.95–38; *p* = 0.05), but not with the progression of WMH. Additional adjustment for clinical SVD manifestations did not alter these associations.^2^ Furthermore, Noz et al. showed that circulating high‐sensitivity IL‐6 (hsIL‐6) highly correlated with WMH at baseline in 2006 and 2015 and with WMH progression between 2006 and 2015 in elderly subjects with mild‐to‐severe CSVD (*p* = 0.005, r_s_ = 0.399; *p* = 0.01, r_s_ = 0.363; *p* = 0.04, r_s_ = 0.294, respectively).^107^

#### 3.3.5 TNF and CSVD

Jefferson and colleagues found that TNF‐α or TNFR2 was not related to WMH/TCV in 1926 Framingham Offspring participants.^104^ Shoamanesh et al. found higher levels of circulating TNFR2 in participants with CMBs (OR 2.2, 95% CI 1.1–4.1) in 1763 stroke‐free Framingham offspring, and a secondary analysis further showed that the association was the most prominent in participants with only deep CMBs. Additionally, the association between TNFR2 and CMBs increased with the increase in CMB burden. As compared with having no CMBs, each unit increase in Ln TNFR2 was associated with a 3.3‐fold increase in the odds of having ≥2 CMBs and 5.7‐fold increase in the odds of having ≥3 CMBs. However, no significant association between TNF‐α levels and prevalent CMB was found.^62^

#### 3.3.6 OPG and CSVD

The role of OPG in CSVD has been poorly studied (Table 1). Jefferson et al. showed that OPG levels were negatively correlated with TCBV (the ratio of total brain volume [TCB] to total cranial volume [TCV]), but not with WMH.^104^ Guldiken and colleagues found that although plasma OPG levels were independently associated with the presence of IS, there was no significant increase in OPG levels in the SVD subtype of stroke.^63^ Shoamanesh et al. observed higher levels of OPG in participants with greater volumes of WMH and/or SCIs in 1763 stroke‐free Framingham offspring. However, since cervical or intracranial large artery disease was not accounted within the study, the results may be secondary to small vessel orifice atheroma or chronic hypoperfusion due to proximal large artery stenosis.^62^

#### 3.3.7 VEGF and CSVD

Zhang et al. showed that elevated VEGF levels were significantly and independently associated with the presence of CMBs in patients with Alzheimer's disease (AD). After controlling for confounding factors, the OR (95% CI) of 10 pg/ml increase in VEGF levels for the presence of CMBs was 2.37 (1.53–4.02) (*p* = 0.004). Multivariate regression analysis further demonstrated a significant correlation between the combination of clinical factors and VEGF levels and the number of CMBs (*p* < 0.001; adjusted R^2^ total = 0.312).^65^

#### 3.3.8 Adhesion molecules and CSVD

##### Adhesion molecules and CSVD prevalence

There is considerable evidence that endothelial activation and dysfunction are associated with CSVD (Table 2). Fassbender et al. found that compared with age‐ and sex‐matched controls, sICAM‐1 was significantly elevated in patients with subcortical vascular encephalopathy (SVE).^68^ Framingham Heart Study found that ICAM‐1 (OR 1.7, 95% CI 1.1–2.5; *p* = 0.02) and lipoprotein‐associated phospholipase A2 (Lp‐PLA2) mass (OR 1.5, 95% CI 1.1–2.1; *p* = 0.01) were positively correlated with extensive WMHs and/or SCIs.^62^ Tchalla et al. observed a linear correlation between the volumes of WMH and the levels of sVCAM‐1 (*r* = 0.47 *p* = 0.018) in community‐based participants, and the association persists significantly after adjusting for comorbidity index.^108^ Similarly, Wu et al. demonstrated that sICAM‐1 levels were associated with the presence of CMBs and increased risk of hemorrhagic transformation (HT) after anticoagulant therapy in 148 patients with acute ischemic stroke (AIS).^109^

**TABLE 2:** Associations of adhesion molecules with MRI features of CSVD

Source	Study design	No.	Filed strength	MRI features	VCAM‐1	ICAM‐1	E‐selectin	P‐selectin
Jefferson et al.^104^	Cross‐sectional	1926	1.0 T	WMHs		No		No
Shoamanesh et al.^62^	Cross‐sectional	1763	1.0 T	CMBs		No		No
				SCI/WMHs		OR = 1.7		No
Tchalla et al.^108^	Cross‐sectional	25	3.0 T	WMHs	*r* = 0.47			
Wu et al.^109^	Cross‐sectional	148	1.5 T	CMBs		Yes		
de Leeuw et al.^69^	Cross‐sectional	29	1.5 T	PVH	Yes	No	No	Yes
				DWMH	No	No	No	No
Han et al.^110^	Cross‐sectional	175	1.5 T	PVH		OR = 5.0		
				DWMH		OR = 4.1		
Rouhl et al.^19^	Cross‐sectional	346	1.5 T	Lacunes	No	No	No	No
				WMHs	No	No	No	No
				CMBs	No	No	*β* = 0.155	No
				BG‐EPVS	No	No	No	No
Huang et al.^111^	Cross‐sectional	126	3.0 T	CMBs			OR = 1.09	
Wang et al.^117^	Cross‐sectional	100	1.5 T	BG‐EPVS		No		
Hassan et al.^70^	Cross‐sectional	110	‐	Lacunes		No		
				WMHs		No		
Markus et al.^113^	Longitudinal (6‐year)	267	1.5 T	WMHs		OR = 1.0		
Kawamura et al.^114^	Longitudinal (3‐year)	120	1.5 T	Lacunes	No	OR = 8.6	No	
				PVH	‐	OR = 2.2	‐	‐
				DWMH		OR = 1.8 (3‐year)		
Umemura et al.^95^	Longitudinal (6‐year)	190	1.5 T	Lacunes		OR = 1.67		
				PVH		OR = 2.17		
				DWMH		OR = 1.83 (3‐year)		
Staszewski et al.^2^	Longitudinal (2‐year)	123	1.5 T	Lacunes		No		No
				WMHs		No		No
Noz et al.^107^	Longitudinal (2‐year)	51	1.5 T	WMHs	No		No	

Abbreviations: BG‐EPVS, basal ganglia‐enlarged perivascular spaces; CMB, cerebral microbleed; DWMH, deep WMH; ICAM‐1, intercellular adhesion molecule 1; OR, odds ratio; PVH, periventricular hyperintensity; SCI, silent cerebral infarct; VCAM‐1, vascular cell adhesion molecule 1; WMH, white matter hyperintensity; *β*/*r*, correlation coefficient.

##### Adhesion molecules and CSVD severity

de Leeuw et al. found that the levels of sP‐selectin and sVCAM‐1 were significantly higher in patients with severe PVH than those with mild. No such association was detected for subcortical DWMH.^69^ However, Han and colleagues demonstrated that sICAM‐1 levels were positively associated with the grade of both subcortical DWMH and PVH in 175 elderly individuals without neurological deficits. Furthermore, multivariate analyses showed that higher sICAM‐1 levels were an independent risk factor for the presence and severity of WMH. After adjusting for other vascular risk factors, an over fourfold increased risk in WMH was found in patients with the highest quartile of sICAM‐1 compared to those with the lowest quartile.^110^ Rouhl et al. and Huang et al. found a positive and significant correlation between sE‐selectin levels and the number of CMBs, irrespective of their location.^19^, ^111^ Rouhl et al. found this association in 163 first‐ever LS patients and 183 essential hypertensive patients, and notably, the data from LS patients were collected approximately 3 months after their stroke to prevent confounding by acute‐phase responses.^19^ Whereas Huang et al. found this association in 126 patients with first‐ever IS, and sE‐selectin levels were measured about 2 weeks after symptom onset.^111^

##### Adhesion molecules and CSVD progression

In a small‐size prospective study, 35 CSVD participants with a mean age of 70 years underwent monthly MRI for 10 consecutive months to detect CSVD progression, defined as any incident lesion (diffusion‐weighted imaging‐positive [DWI+] lesion, microbleed, or lacune) and the first quartile of WMH progression. Totally, 13 out of 35 participants had CSVD progression with either incident lesions (*n* = 7) and/or the upper quartile of WMH progression (*n* = 9). E‐selectin levels were higher in participants with CSVD progression than those without (19.4 [16.8–24.3] vs. 13.9 [10.7–17.5] pg/ml; *p* < 0.05).^112^ The community‐based Austrian Stroke Prevention Study found that ICAM‐1 was associated with age‐ and gender‐adjusted WMH lesion progression at both 3 and 6 years (3‐year OR, 1.007; 95% CI, 1.002–1.012; *p* = 0.004; and 6‐year OR, 1.004; 95% CI, 1.000–1.009 per ng/ml; *p* = 0.057). After adjusting for other cardiovascular risk factors and CRP, these associations persisted significantly with 3‐year OR 1.010 (95% CI, 1.004–1.017; *p* = 0.001) and 6‐year OR 1.008 (1.002–1.014 per ng/ml; *p* = 0.006).^113^ A 3‐year longitudinal observational study revealed that higher levels of sICAM‐1 were an independent risk factor for SCI progression in patients with type 2 diabetes mellitus (T2DM). The relative risk for SCI progression over 3 years was around eight times in the highest quartile of sICAM‐1 levels at baseline as compared with the lowest quartile after adjusting for covariables (OR 8.61, 95% CI 2.04–36.3; *p* < 0.005).^114^ A 6‐year longitudinal study included a total of 190 patients with T2DM (mean age 62.7 years; mean duration of diabetes 13.1 years) who were free of dementia and assessed the association between baseline sICAM‐1 levels and progression of SBIs and periventricular and subcortical WMHs on MRI at 3 and 6 years. Logistic regression analysis showed that the ORs (95% CI) associated with a 1SD increase in baseline sICAM‐1 levels were 1.99 (1.29–2.80) for SBI progression, 2.36 (1.30–4.30) for periventricular WMH progression, 1.83 (1.19–2.81) for subcortical WMH progression at the 3‐year follow‐up, and 1.67 (1.02–3.05) for SBI progression and 2.17 (1.29–3.62) for periventricular WMH progression at the 6‐year follow‐up. These associations remain significant after controlling for age, sex, hypertension, and duration of diabetes, baseline MRI findings, and medication use.^95^

#### 3.3.9 Hemostasis factors and CSVD

The associations of hemostasis factors with MRI features of CSVD have been studied, but the results are inconsistent (Table 3). Among 160 asymptomatic, high‐risk Japanese subjects who were at least 60 years of age, 84 (53%) had > or = 1 lacunar infarcts (silent lacunar group) and the remaining 76 had no lacunar infarct (nonlacunar group). The levels of fibrinogen and F 1 + 2 were significantly higher in the former than those in the latter (*p* < 0.01). When further classifying the silent lacunar group into three subgroups according to the number of lacunes (few lacunes, 1 or 2; moderate number of lacunes, 3 or 4; and numerous lacunes, > or = 5), levels of F 1 + 2, vWF, and TM in the numerous lacunes were significantly higher than those in the few‐lacunes subgroup.^115^ Similarly, in a study including 123 asymptomatic elderly hypertensive subjects, multiple lacunar infarcts were significantly independently associated with vWF, PAI‐1 and F1 + 2, and basal ganglia infarcts were associated with PAI‐1 and D‐dimer.^73^ In a nested sample from a subset of the Atherosclerosis Risk in Communities (ARIC) cohort, Gottesman found that vWF and D‐dimer were positively associated with silent lacunar infarcts.^116^ However, in a study involving 100 patients with lacunar or minor cortical IS, vWF was found to be negatively associated with BG PVS count (*p* = 0.032). The authors suggested that vWF may promote cerebral endothelial integrity, while lack of vWF may indicate cerebral endothelial dysfunction, increased BBB permeability, and increased BG PVS.^117^ Hassan et al. found elevated levels of ICAM‐1, TM, and TFPI in white Caucasian patients with CSVD. The ischemic LA group had a lower TFPI level (*p* = 0.01) and a higher TF/TFPI ratio (*p* = 0.01) compared with the isolated lacunar infarction group. Besides, TM level was observed to be positively correlated with both the number of lacunes (*p* = 0.008) and the extent of LA (*p* = 0.03), whereas TF level and the TF/TFPI ratio were associated only with the extent of LA (*p* = 0.02 and 0.01).^70^

**TABLE 3:** Associations of hemostasis factors with MRI features of CSVD

Source	Study design	No.	Filed strength	MRI features	D‐Dimer	vWF	TM	F 1 + 2	TF	TFPI	TAT	t‐PA	PAI‐1
Wada et al.^97^	Cross‐sectional	667	‐	Lacunes		No	No						
				WMHs		No	No						
Liu et al.^102^	Cross‐sectional	85	3.0 T	CMBs	No								
Kario et al.^115^	Cross‐sectional	160	1.5 T	Lacunes		No	No	Yes					
Kario et al.^73^	Cross‐sectional	123	1.5 T	Lacunes	No	OR = 5.7		OR = 5.6			No		OR = 13.0
Gottesman et al.^116^	Cross‐sectional	410	1.5 T	Lacunes	OR = 1.76	OR = 2.04	No					No	No
Wang et al.^117^	Cross‐sectional	100	1.5 T	BG‐EPVS	No	*r* = −0.25		No			No	No	
Hassan et al.^70^	Cross‐sectional	110	‐	Lacunes			Yes		No	No			
				WMHs			Yes		Yes	No			
Markus et al.^113^	Longitudinal. (6‐year)	267	1.5 T	WMHs	No		No	No		No			
Staszewski et al.^103^	Longitudinal. (2‐year)	123	1.5 T	Lacunes	No		No		No				
				WMHs	No		No		No				

Abbreviations: BG‐EPVS, basal ganglia‐enlarged perivascular spaces; CMB, cerebral microbleed; F1 + 2, prothrombin fragment 1 + 2; OR, odds ratio; PAI‐1, plasminogen activator inhibitor‐1; *r*, correlation coefficient; TAT, thrombin‐antithrombin complex; TF, tissue factor; TFPI, tissue factor pathway inhibitor; TM, thrombomodulin; t‐PA, type plasminogen activator; vWF, von Willebrand factor; WMH, white matter hyperintensity.

#### 3.3.10 Homocysteine and CSVD

##### Homocysteine and CSVD prevalence

Results of studies on the relationship between Hcy and MRI features of CSVD are inconsistent (Table 4). In the Cardiovascular Health Study, plasma tHcy levels were not associated with individual MRI findings of white matter grade, or infarcts in 622 elderly subjects without a history of TIA or stroke. However, a linear trend was found between quintiles of tHcy level and an MRI pattern combining infarcts and high white matter grade. The linear trend remained significant after adjustment for other risk factors and atherosclerotic markers but was slightly diminished after further adjustment for creatinine, folate, and vitamins B6 and B12.^118^ Matsui and colleagues showed that the elevated tHcy levels were significantly correlated with SBI after individual adjustment for age, sex, hypertension, renal function, and the habits of smoking and alcohol consumption in community‐dwelling elderly people.^119^, ^120^

**TABLE 4:** Association of plasma Hcy levels with MRI features of CSVD

Source	Study design	No. of Subjects	Field strength (Tesla)	MRI features	Hcy
Shoamanesh et al.^62^	Cross‐sectional	1763	1.0 T	CMBs	No
				SCIs/WMHs	No
Longstreth et al.^118^	Cross‐sectional	622	–	Lacunes	No
				WMHs	No
Matsui et al.^119^	Cross‐sectional	153	1.0 T	SCIs	OR = 4.5
Vermeer et al.^121^	Cross‐sectional	1077	1.5 T	Lacunes	OR = 2.5
				WMHs	OR = 2.3
Hassan et al.^17^	Cross‐sectional	172	–	Lacunes	OR = 12.9
				WMHs	OR = 4.2
Naka et al.^122^	Cross‐sectional	102	1.0 T	WMHs	OR = 1.3
				CMBs	No
Pavlovic et al.^124^	Cross‐sectional	95	1.0 T	WMHs	OR = 1.2
Feng2013^125^	Cross‐sectional	324	1.5 T	Lacunes	*r* = 0.339
				WMHs	*r* = 0.379
Gao et al.^126^	Cross‐sectional	923	–	WMHs	OR = 1.9
Miwa et al.^127^	Cross‐sectional	643	–	Lacunes	OR = 1.8
				WMHs	No
				CMBs	OR = 2.0
				Atrophy	No
Wright et al.^128^	Cross‐sectional	259	1.5 T	WMHs	OR = 4.2
Wong et al.^74^	Cross‐sectional	57	1.5 T	Lacunes	No
				WMHs	R^2^ = 0.06
				Atrophy	No
Seshadri et al.^129^	Retrospective	1965	–	Lacunes	RR = 1.5
				WMHs	No
				Atrophy	Yes
Raz et al.^130^	Cross‐sectional	144	4 T	WMHs	No
Narayan et al.^131^	Longitudinal (2‐year)	70	1.5 T	WMHs	No
				Atrophy	*β* = 0.46
Kloppenborg et al.^132^	Longitudinal (4‐year)	663	1.5 T	Lacunes	No
				WMHs	OR = 2.4
Staszewski et al.^2^	Longitudinal (2‐year)	123	1.5 T	Lacunes	OR = 1.9
				WMHs	No

Abbreviations: CMB, cerebral microbleed; Hcy, homocysteine; OR, odds ratio; SCI, silent cerebral infarct; WMH, white matter hyperintensity; *β*/*r*, correlation coefficient.

##### Homocysteine and CSVD severity

Several studies have clearly demonstrated the positive association between tHcy levels and the severity of MRI lesions in patients with CSVD. Vermeer et al. showed that tHcy levels are associated with SBIs and WMHs independent of each other and of other cardiovascular risk factors. Subjects were 24% more likely to have SBIs per SD increase in tHcy (95% CI 6%–45%). The severity of periventricular WMHs and the extent of subcortical WMHs also significantly correlated with tHcy levels, even after the exclusion of those with SBI. Further, the overall risk of having either an SBI or severe WMH increased by 35% for each SD increase in tHcy (95% CI 16%–58%).^121^ Hassan et al. found that Hcy is an independent risk factor for CSVD, especially for ischemic LA subtype, and that the risk of CSVD increased with increasing quartile of Hcy. After adjusting conventional risk factors and creatinine, the OR for CSVD associated with Hcy was 8.34 (95% CI 3.63 ± 19.14) per 1 μmol increase in log concentration (*p* < 0.0005). The OR was only slightly reduced (to 7.91, 95% CI 3.93 ± 18.44; *p* < 0.0005) after additional adjustment for MTHFR genotype. They also confirmed that Hcy levels correlated with ICAM‐1 and TM, and the addition of these markers as covariates reduced the association between Hcy levels and SVD but improved the predictive model for the presence of SVD, suggesting that the effect of Hcy on SVD may be mediated by endothelial dysfunction.^17^ Naka and colleagues revealed that increased tHcy levels are significantly and independently associated with advanced LA (early confluent or confluent LA; OR, 1.330; 95% CI, 1.130–1.565) but not with the presence of microbleeds in patients with stroke, indicating that elevated tHcy levels appear to be associated with ischemic SVD rather than with bleeding‐prone SVD.^122^ Ma et al. showed that tHcy level was positively associated with the LA severity in SVD (*r* = 0.308, *p* = 0.001). After adjustment for age, gender, vascular risk factors, and eGFR, mean tHcy level was significantly higher in patients with moderate (*p* < 0.001) and severe (*p* < 0.001) LA compared with patients without it.^123^ Pavlovic and colleagues showed that the elevated tHcy level was positively and independently correlated with the severity of WMH in Serbian patients with SVD.^124^ Feng and colleagues showed that, in 324 nonstroke patients, Hcy level was positively correlated with scores of LA (*r* = 0.379, *p* < 0.001) and numbers of SBI (*r* = 0.339, *p* < 0.001). Additionally, the authors demonstrated that Hcy level was an independent risk factor for SVD (OR = 1.315, *p* < 0.001).^125^ Gao et al. showed that in patients with acute ischemic stroke (mean age, 58.9 ± 11.9 years; female, 31.6%), elevated plasma tHcy level was significantly and independently related to WMHs severity. For the highest tHcy quartile, the OR (95% CI) was 1.891 (1.257; 2.843) according to the Fazekas scale and 1.781 (1.185; 2.767) according to the age‐related white matter changes (ARWMC) visual grading scale when compared to the lowest quartile. However, subgroup analysis further showed that only WMHs distributed within the periventricular area and left or right frontal areas were independently associated with tHcy level.^126^ Miwa and colleagues observed the cross‐sectional association between the highest tHcy tertile and lacunes, CMBs, as well as strictly deep CMBs within a Japanese cohort of participants with vascular risk factors, after the adjustment for several potential confounders. Although higher tHcy levels were associated with PVH and DWMH when adjusted for age and gender only, the correlation disappeared after further adjustment for age, gender, BMI, MMSE, smoking, hypertension, previous cerebrovascular events, eGFR, and IMT.^127^ In a stroke‐free community‐based population of Hispanic, black, and white participants, Wright et al. found that tHcy levels were associated with pixel‐based quantitative measures of WMHV. Log‐tHcy level and the highest tHcy category compared with the lowest both correlated with log‐WMHV and WMHV‐large (WMHV >1 SD). The association remained significant after adjusting for age, sex, race‐ethnicity, hypertension, cardiac disease, and B12 deficiency.^128^ In a small‐size (*n* = 57), prospective, cohort‐based study, HHcy, defined as the highest (4th) quartile of Hcy level, significantly accounted for the WMHV in a multivariate stepwise liner regression model after adjusting for age and folate level. However, no significant association was seen between Hcy levels and SBI, brain atrophy as well as cognitive functions.^74^ In a community‐based sample of middle‐aged adults who were free of clinical stroke, dementia, or other neurologic disease affecting brain MRI and had at least 1 measurement of plasma tHcy level between 1991 and 2001 as well as a brain MRI between 1999 and 2002, subjects with a plasma tHcy level in the highest age‐ or sex‐specific quartile had smaller total cerebral brain volumes (TCBV), the ratio of total brain parenchymal volume to total cranial volume, compared to those with lower tHcy levels. More importantly, initial (1991–1995) plasma tHcy levels were associated with a higher prevalence of SBI (RR, 1.5; 95% CI, 1.1–2.1; *p* = 0.02), and concurrent (1998–2001) plasma tHcy levels were associated with smaller frontal (−0.14%, *p* = 0.001) and temporal lobar (−0.10%, *p* = 0.04) volumes. However, there was no significant association between the prevalence of extensive WMH and initial or concurrent plasma tHcy levels.^129^ Raz and colleagues suggested that, in healthy adults who are free of neurological and vascular disease, tHcy had no effect on WMHV, but the interaction between tHCy and age was related to WMHV. The strength and shape of this association varied across the cerebral lobes. In the frontal, parietal, and temporal lobes, WMHV was the largest in the elderly with high tHcy levels, while in the occipital lobes, the greatest WMHV was seen in the middle‐aged with high tHcy.^130^

##### Homocysteine and CSVD progression

Narayan et al. have demonstrated that plasma tHcy levels significantly correlated with increased rates of whole‐brain atrophy but not WMH progression by using serial MRI in a community‐dwelling population with hypertension with an average age of 79.1 years. The association between tHcy levels and brain atrophy rates persisted even after adjusting potential confounders such as folate and B12, which are known to alter tHcy levels and others that could influence brain atrophy, such as age, BP, and serum creatinine. However, there was no correlation of total, periventricular or deep WMH load, or their rate of progression with tHcy levels on simple correlation studies as well as on multivariate analysis. In addition, new SBIs were uncommon with only one subject developing a new small cortical infarct during the 2‐year inter‐MRI study interval. However, the results of the study may be not convincing enough because of the small size with only 70 subjects.^131^ In a large prospective longitudinal cohort study of 663 independently living patients with symptomatic atherosclerotic disease with a mean follow‐up of 3.9 years, Kloppenborg et al. found that, after controlling for age, sex, and follow‐up time, HHcy (highest quintile of Hcy vs. lower 4 quintiles) was significantly associated with increased risk of WMH progression (OR 2.4, 95% CI 1.4–3.9, *p* = 0.001) and of new lacunar infarcts (OR 1.9, 95% CI 1.1–3.6, *p* = 0.031). After accounting for vascular risk factors and IMT, the association between HHcy and WMH progression remained significant (OR 2.4, 95% CI 1.5–4.1), while the association between HHcy and new lacunar infarcts disappeared (OR 1.8, 95% CI 0.9–3.4).^132^

#### 3.3.11 Composite factors

Some studies tried to use composite factors to reflect the inflammation process in the development and progression of CSVD and obtained encouraging outcomes. A population‐based study of 268 elderly individuals showed that single pro‐ (IL‐1β, IL‐6, IL‐8, IL‐12, and TNF‐α) and anti‐inflammatory (sIL‐4R anf IL‐10) cytokines were not associated with subcortical WMH, atrophy, or lacunar infarctions, after the Bonferroni correction for multiple comparisons. However, there was a significant association between atrophy and the chemokine‐cytokine factor (a composite measure of sIL‐4R, IL‐6, and IL‐8), even after controlling for confounders.^133^ Similarly, a single‐center prospective cohort study indicated that the Z‐score for vascular inflammation (containing sICAM‐1, sP‐selectin, sCD40 L, platelet factor‐4 [PF‐4], and Hcy) was significantly related to WMHs progression or new lacunes; while, Z‐score for systemic inflammation (containing hs‐CRP, IL‐1α, IL‐6, and TNF‐α) was correlated with the development of new lacunes. These associations persist significantly after additional adjustment for clinical CSVD manifestations.^2^ Recently, Altendahl et al. identified a cross‐sectional relationship between an IL‐18‐centered systemic inflammatory network and antecedent and overt white matter injury in two distinct populations from the MarkVCID study and ASPIRE study cohorts, respectively. The authors demonstrated that the inflammation composite score (ICS), a composite measure of inflammatory markers (myeloperoxidase [MPO], growth differentiation factor 15 [GDF‐15], receptor for advanced glycation end products [RAGE], ST2, interleukin‐18 [IL‐18], and monocyte chemoattractant protein‐1 [MCP‐1]), was significantly associated with log WMH (*β* = 0.222, *p* = 0.013) as well as DTI FW (*β* = 0.3, *p* = 0.01).^134^ Homoplastically, Kuipers and colleagues assessed the relation between 92 blood‐based biomarkers from the OLINK cardiovascular III panel and CSVD and identified a cluster of biomarkers reflecting coagulation, which is related to the manifestations of CSVD, including WMHs, lacunar infarcts, CMBs, and EPVS. In addition, the authors found a mediation effect of the biomarker cluster on the relation between age and WMH ratio (proportion mediated 17%), and hypertension and WMH volume (proportion mediated 21%). Therefore, the authors suggested the involvement of coagulation abnormalities in the etiology of CSVD.^135^ In conclusion, these studies suggest that the composite measures of multiple functional‐interrelated biomarkers may be more relevant to CSVD than individual biomarkers.

## 4 CONCLUSIONS

In conclusion, although there are conflicting results from different studies, the existing studies of interest suggest that inflammation may play an important role in the development and progression of CSVD. Compared with individual plasma biomarkers, clusters of interrelated biomarkers associated with CSVD may better explain the underlying pathological processes.

### 4.1 Limitations and future directions

This article has some limitations: First, the detailed mechanisms of inflammation on CSVD are not well described in this article. Second, not all the research on the relationship between inflammatory biomarkers and MRI features of CSVD is included. Last but not least, studies evaluating the inflammatory biomarkers with clinical endpoints of CSVD, such as stroke and cognitive decline, are not included. With the development of advanced MRI technology, the pathological mechanisms underlying CSVD may be further revealed and early diagnosis, and even reversal, of CSVD may be possible in the future. Besides, clusters of interrelated biomarkers may be more relevant to CSVD than individual biomarkers; thus, further studies focusing on biomarker clusters may be helpful.

## AUTHOR CONTRIBUTIONS

SW drafted this manuscript. GH and YG searched, read, and organized literature. CD, YD, HS, and RM revised the manuscript and edited English. RM contributed to the conception and design of this study and proposed the amendments.

## FUNDING INFORMATION

This work was supported by the National Natural Science Foundation of China (Grant/Award Number: 81371289 and 82101390) and the Natural Science Foundation of Beijing Municipality (Grant/Award Number: 7212047).

## CONFLICT OF INTEREST

The authors declare that this work has no conflict of interest.

## Data Availability

Data sharing is not applicable to this article as no new data were created or analyzed in this study.
